# The potential renal acid load of plant-based meat alternatives

**DOI:** 10.1038/s41430-024-01434-8

**Published:** 2024-03-19

**Authors:** Julian Herter, Roman Huber, Maximilian Andreas Storz

**Affiliations:** Department of Internal Medicine II, Centre for Complementary Medicine, Freiburg University Hospital, Faculty of Medicine, University of Freiburg, Freiburg im Breisgau, Germany

**Keywords:** Nutrition, Public health

## Abstract

Plant-based meat alternatives (PBMAs) are food products derived from plants and designed to mimic the preparation methods, nutritional profile, and sensorial qualities of meat. PBMAs are currently subject to a controversial debate concerning their health value. Here, we reviewed PBMAs’ potential renal acid load (PRAL). The PRAL is an estimate for the amount of acid or base a certain food produces in the human body, and was associated with tissue damage and acid stress. PRAL values varied substantially across the examined foods, and differences were as large as 19.73 mEq per 100 g of PBMA. Mycoprotein- and wheat-based PBMAs were more acidic than conventional meats. The majority of items, however, exerted a lower PRAL to the human kidneys when compared to their meat-based counterparts. Our findings reiterate that not all PBMAs are created equal, and suggest that PBMAs are generally not suitable to substantially alkalize an individual’s diet.

## Introduction

Plant-based diets enjoy growing popularity in many Western countries [1]. As opposed to ‘traditional’ whole-food plant-based diets, the fast-paced nature of contemporary lifestyles has drastically increased the demand for plant-based convenience foods [2]. Plant-based meat alternatives (PBMAs) are commercially available food products, which were derived from plant, and which were designed to mimic the preparation methods, sensorial qualities and nutritional profile of meat-based equivalents (MBEs).

As novel food items, PBMAs are subject to a controversial debate concerning their health value [2, 3]. This debate mainly focused on nutritional profiles but has rarely covered PBMAs’ acid-based impact [4].

The Potential Renal Acid Load (PRAL) score is an estimate for the amount of acid or base a certain food produces in the body [1]. High-PRAL foods exert a substantial acid load to the human kidneys, and may promote tissue inflammation and low-grade acidosis when consumed over a long time. They were also associated with cardiovascular disease, diabetes and altered cortisol metabolism in numerous studies [1].

Based on the current knowledge of PBMAs’ nutrient profiles [2], we hypothesized that PBMAs would differ in PRAL scores in comparison to their MBEs. To test this hypothesis, we performed a secondary data analysis and estimated the PRAL value of the most commonly consumed PBMAs.

## Materials and methods

### Food item collection

This brief contribution builds on published data from other sources. We used PubMed and Google Scholar to identify scientific articles that investigated the nutrient content of PBMAs. We performed search queries with combinations of the following keywords: “meat analog”, “plant-based meat”, “meat alternative”, “nutrient content”, and “nutritive value”. For PRAL estimation, nutrient content data of the following nutrients was required: protein, magnesium, potassium, calcium and phosphorus. Thus, only sources that covered these particular nutrients were considered. Based on our criteria, we identified 3 articles from which we extracted data [5–7]. For reference purposes, we calculated the PRAL value of common meats using data from FoodData Central [8].

### PRAL estimation

Our methods for PRAL estimation have been described in related publications [9]. In brief, we used the formula by Remer and Manz to calculate PRAL in mEq/100 g portions [10]. PRAL corrects for intestinal absorption of ingested minerals and sulfur-containing protein, and takes into account ionic dissociation [10]. Food items with a PRAL value > 0 exert acidifying properties, whereas a PRAL value < 0 indicates alkalizing effects [1].

PRAL = (0.49 * protein intake (g/100 g)) + (0.037 * phosphorus intake (mg/100 g)) - (0.021 * potassium intake (mg/100 g)) - (0.026 * magnesium intake (mg/100 g)) - (0.013 * calcium intake (mg/100 g))

### Statistical analysis

We described relevant statistical procedures elsewhere in detail [9]. Data was analyzed with STATA 14 statistical software. Based on Stata’s Shapiro–Wilk test, we decided whether data was normally distributed or not. Pearson’s product-moment correlations were run to assess the relationship between nutrient contents and PRAL.

## Results

As part of the secondary data analysis of Harnack et al. [5], we analyzed the PRAL value of *n* = 37 plant-based ground beef alternatives (Table 1). PRAL values varied substantially across the examined foods, and ranged from −2.89 mEq/100 g to 16.84 mEq/100 g. The mean PRAL value was 4.04 ± 4.73 mEq/100 g and thus suggested a moderately acidifying potential (PRAL > 0 mEq/d). Almost 80% of the examined items had a PRAL value < 8 mEq/100 g, and thus ranked lower than conventional pork and beef meat, which both have PRAL values of approximately 8 mEq/100 g.

**Table 1:** Nutrient content and PRAL values of selected plant-based ground beef alternative products in the United States.

Product name	Producer	Vegan	Protein	Ca	K	Mg	P	PRAL
All American Veggie Burger	Amy’s Kitchen, Inc.	Yes	15.88	61.18	276.47	54.35	191.18	6.84
Organic Black Bean Veggie Burger	Amy’s Kitchen, Inc.	Yes	8.47	30.59	442.35	54.35	161.76	−0.96
Organic California Veggie Burger	Amy’s Kitchen, Inc.	Yes	8.82	45.88	331.76	64.24	191.18	2.16
Organic California Veggie Burger, Light in Sodium	Amy’s Kitchen, Inc.	Yes	8.82	45.88	331.76	64.24	191.18	2.16
Organic Sonoma Veggie Burger, Gluten Free, Dairy Free	Amy’s Kitchen, Inc.	Yes	6.24	30.59	331.76	64.24	176.47	0.55
Organic Summer Harvest Veggie Burger	Amy’s Kitchen, Inc.	Yes	5.65	61.18	276.47	69.18	147.06	−0.19
Quarter Pound Veggie Burger	Amy’s Kitchen, Inc.	Yes	17.29	76.47	331.76	49.41	161.76	5.21
Beyond Beef	Beyond Meat	Yes	18.00	15.29	276.47	34.59	147.06	7.36
Beyond Beef Crumbles Beefy	Beyond Meat	Yes	22.00	15.29	55.29	49.41	235.29	16.84
Beyond Burger	Beyond Meat	Yes	18.00	15.29	276.47	34.59	147.06	7.36
Gardein Beefless Ground	Conagra, Inc.	Yes	21.18	107.06	718.82	39.53	250.00	2.11
Impossible Burger	Impossible Foods Inc.	Yes	16.94	152.94	552.94	9.88	161.76	0.43
Gardenburger Black Bean Chipotle Veggie Burgers	Kellogg NA Co.	Yes	6.94	45.88	221.18	44.47	117.65	1.36
Gardenburger Original Burgers	Kellogg NA Co.	No	6.82	61.18	165.88	44.47	191.18	4.98
Gardenburger Portabella Veggie Burgers	Kellogg NA Co.	No	5.53	45.88	221.18	54.35	147.06	1.50
Morningstar Farms Cheezeburger	Kellogg NA Co.	Yes	20.71	91.76	165.88	9.88	132.35	10.11
Morningstar Farms Chipotle Black Bean Crumbles	Kellogg NA Co.	Yes	13.65	45.88	221.18	74.12	117.65	3.87
Morningstar Farms Falafel Burgers	Kellogg NA Co.	Yes	7.65	76.47	221.18	34.59	117.65	1.56
Morningstar Farms Garden Veggie Burgers	Kellogg NA Co.	No	16.35	91.76	221.18	39.53	88.24	4.41
Morningstar Farms Grillers Crumbles	Kellogg NA Co.	Yes	14.82	107.06	442.35	88.94	205.88	1.89
Morningstar Farms Grillers Original Veggie Burgers	Kellogg NA Co.	No	25.65	91.76	165.88	24.71	73.53	9.97
Morningstar Farms Grillers Prime Veggie Burgers	Kellogg NA Co.	No	22.59	61.18	165.88	19.76	117.65	10.63
Morningstar Farms Meat Lovers Vegan Burgers	Kellogg NA Co.	Yes	24.12	30.59	165.88	24.71	161.76	13.28
Morningstar Farms Mediterranean Chickpea Burgers	Kellogg NA Co.	No	15.65	76.47	276.47	44.47	102.94	3.52
Morningstar Farms Roasted Garlic & Quinoa Burgers	Kellogg NA Co.	Yes	10.71	61.18	552.94	44.47	147.06	−2.89
Morningstar Farms Spicy Black Bean Burgers	Kellogg NA Co.	No	13.06	76.47	387.06	59.29	117.65	0.09
Morningstar Farms Spicy Indian Veggie Burgers	Kellogg NA Co.	Yes	9.53	61.18	497.65	29.65	132.35	−2.45
Morningstar Farms Tex-Mex Burgers	Kellogg NA Co.	Yes	9.53	76.47	387.06	54.35	132.35	−0.97
Morningstar Farms Tomato & Basil Pizza Burgers	Kellogg NA Co.	No	16.59	137.65	331.76	34.59	161.76	4.46
Morningstar Farms Veggie Lovers Vegan Burgers	Kellogg NA Co.	Yes	7.88	30.59	276.47	44.47	132.35	1.40
Morningstar Farms White Bean Chili Veggie Burgers	Kellogg NA Co.	Yes	11.88	91.76	552.94	49.41	147.06	−2.83
BOCA All American Veggie Burgers	Kraft Foods, Inc.	No	18.71	137.65	165.88	19.76	191.18	10.45
BOCA Original Veggie Crumbles	Kraft Foods, Inc.	Yes	19.65	122.35	110.59	34.59	191.18	11.89
Quorn Meatless Grounds	Marlow Foods Ltd.	No	13.88	45.88	110.59	19.76	29.41	4.46
Quorn Vegan Meatless Spicy Patties	Marlow Foods Ltd.	Yes	12.24	30.59	110.59	19.76	29.41	3.85
Plant-Based Crumbles Beef Style	Tofurky	Yes	16.71	91.76	608.24	93.88	220.59	−0.06
Worthington Meatless Fripat	Worthington	No	22.00	122.35	442.35	9.88	147.06	5.08

Data obtained and modified from Harnack et al. [5]. Nutrient content per 100 g cooked portions; PRAL value for a 100 g edible portion. PRAL in mEq/100 g. Protein in g/100 g. Calcium (Ca), Potassium (K), Magnesium (Mg) and Phosphorus (P) in mg/100 g. Note: Harnack et al. [5] reported nutrient content in mg and g/3 ounce cooked portions, which was used here to calculate nutrient contents of 100 g portions.

The protein content per 100 g cooked portion ranged from 5.53 g to 25.65 g and was positively associated with PRAL (*r* = 0.73, *p* < 0.001). Significant inverse associations with PRAL were found for the magnesium (*r* = −0.41, *p* = 0.01) and potassium content (*r* = −0.69, *p* < 0.001). The “Beyond Beef Crumbles Beefy” and the “Morningstar Farms Meat Lovers Vegan Burgers” were the most acidifying food items, with PRAL values of 16.84 and 13.28 mEq/100 g, respectively.

**Fig. 1:**
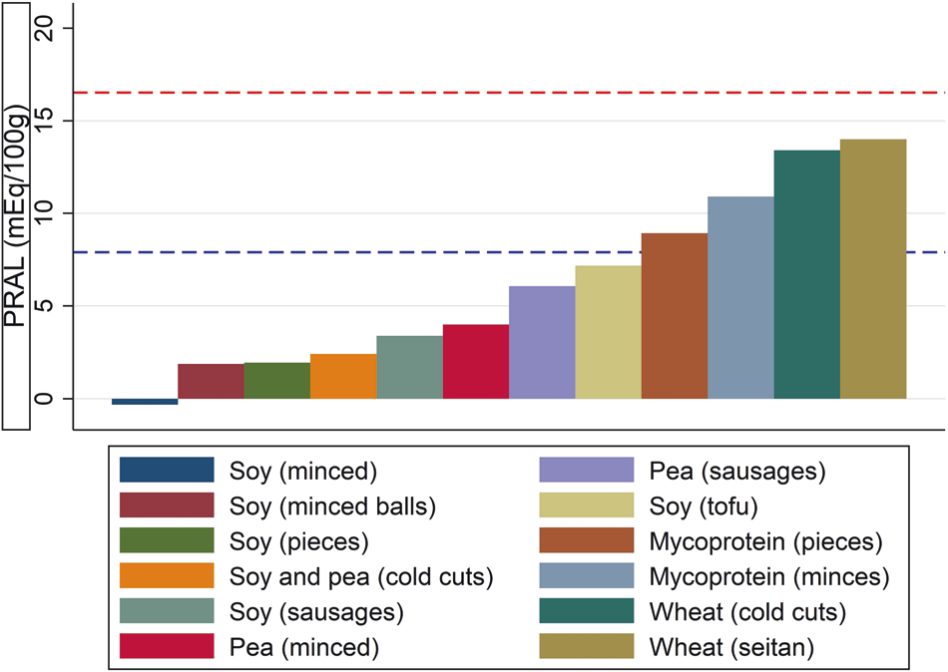
PRAL values of selected plant-based meat alternative groups. legend: based on data from Ložnjak Švarc et al. [6]. Data based on a nutrient analysis of *n* = 58 products with protein derived from egg white, mycoprotein, pea, soy, a combination of pea and soy, and wheat. PRAL in mEq/100 g. The blue dotted line indicates the PRAL value of a 100 g portion of beef, whereas the red dotted line indicates the PRAL value of a 100 g portion of chicken.

Further to that, we used data from Ložnjak-Švarc, who performed a nutrient analysis of *n* = 58 products with protein derived from egg white, mycoprotein, pea, soy, a combination of pea and soy, and wheat [6]. Readily available on the Danish food market, these products were divided into several groups, such as cold cuts, minced, or sausages. We adopted this classification and calculated group-specific PRAL values (Fig. 1). The mean PRAL value in this food sample was 6.15 ± 4.76 mEq/100 g. The mycoprotein- and wheat-based food items yielded the highest PRAL values, ranging from 8.92 to 13.99 mEq/100 g.

Finally, we analyzed data from De Marchi et al., who compared selected Italian plant-based and meat-based burgers (Supplementary Table 1) [7]. The PRAL values between both groups did not differ substantially (6.80 vs. 7.27 mEq/100 g), probably because of the rather similar protein content of the examined items. While plant-based burgers were more abundant in potassium (an alkali precursor), they also included more acidifying phosphorus.

## Discussion

Our results suggest a large heterogeneity in terms of PBMAs’ acid-based impact, and reiterate that not all PBMAs are created equal. PRAL values varied substantially across foods, and differences were as large as 19.73 mEq/100 g. In comparison to their MBEs, some mycoprotein-based PBMAs were more acidic. The majority of foods, however, was more alkaline when compared to beef and pork.

PBMAs were designed to mimic the nutritional profile of MBEs [2, 3]. Inherent to their purpose, they are processed foods with a moderate-to-high protein content. Thus, a positive PRAL value is naturally to be expected. Yet, differences in PRAL-relevant micronutrients could play a pivotal role. One example is potassium, which appears to be more abundant in PBMAs [7]. At the same time, some PBMAs also contain more phosphorus [7], which contributes to their acidity [9].

High-PRAL diets are associated with cardiovascular and kidney disease [1]. Selecting low-PRAL foods may thus be important for some individuals. In the case of PBMAs, this appears difficult, as labeling of mineral information on packaged food labeling is at the discretion of the manufacturer in the European Union [2]. Data on micronutrient content is often lacking, and our PRAL-table might thus be helpful for individuals who wish to adopt a more alkaline diet. The original PRAL tables by Remer and Manz were constructed before the emergence of PBMAs, and thus do not contain this novel food group. Inherent to a secondary data analysis, however, we did not measure nutrient contents of the examined foods ourselves, and acknowledge that methods and/or validity may have deviated between the examined datasets.

Some evidence suggests that PBMAs might have a healthier nutrient profile than MBEs [11]. While the PRAL value of most items might be slightly lower, it is not anywhere near the (negative) PRAL value of unprocessed high-protein plant foods (e.g., beans). While PBMAs could offer a steppingstone in the transition away from meat to increased plant consumption, they might be unsuitable to substantially alkalize an individual’s diet.

### Supplementary information

Supplementary Table 1

Supplementary Table 2

## Data Availability

The datasets used and analyzed during the current study are available from the corresponding author on reasonable request.
