# Supplementary material for: The potential renal acid load of plant-based meat alternatives
Source: Eur J Clin Nutr. 2024 Mar 19;78(8):732–5. doi: 10.1038/s41430-024-01434-8 (PMC11300296; doi:10.1038/s41430-024-01434-8)
Supplement: Supplementary file 1 — Supplementary Table 1 [file 41430_2024_1434_MOESM1_ESM.docx]

# Supplementary Table 1

Supplementary Table 1 title: PRAL values and median nutrient content of meat-based and plant-based burgers: a comparison.

| **Product** | **Protein** | **Ca** | **K** | **Mg** | **P** | **PRAL** |
| --- | --- | --- | --- | --- | --- | --- |
| Meat-based burgers | 17.96 | 8.56 | 271.76 | 15.94 | 127.01 | 7.27 |
| Plant-based burgers | 18.01 | 71.54 | 345.71 | 61.49 | 209.89 | 6.80 |

Supplementary Table 1 legend: modified from De Marchi et al. [7]. PRAL in mEq/100g. Protein in g/100g. Calcium (Ca), Potassium (K), Magnesium (Mg) and Phosphorus (P) in mg/100g. The plant-based burger group included the following items: Beyond burger (Beyond Meat, USA; Grocery store Metro di Padova, Padova, Italy), Incredible burger (Garden Gourmet, Nestlé, Switzerland; Grocery store Esselunga, Padova, Italy), and Planty of burger (Planty of meat, Frostmeat GmbH, Germany; Grocery store Metro di Padova, Padova, Italy).
