# Supplementary material for: The potential renal acid load of plant-based meat alternatives
Source: Eur J Clin Nutr. 2024 Mar 19;78(8):732–5. doi: 10.1038/s41430-024-01434-8 (PMC11300296; doi:10.1038/s41430-024-01434-8)
Supplement: Supplementary file 2 — Supplementary Table 2 [file 41430_2024_1434_MOESM2_ESM.docx]

# Supplementary Table 2

Supplementary Table 2 title: The PRAL value of selected meats

| **Product** | **Protein** | **Ca** | **K** | **Mg** | **P** | **PRAL** |
| --- | --- | --- | --- | --- | --- | --- |
| Beef, ground, 90% lean meat / 10% fat, raw | 18.2 | 7 | 281 | 16.5 | 148 | 7.97 |
| Pork, ground, raw | 17.8 | 6 | 318 | 19 | 173 | 7.87 |
| Chicken, broiler or fryers, breast, skinless, boneless, meat only, cooked, braised | 32.1 | 6 | 343 | 32 | 241 | 16.53 |

Supplementary Table 2 legend: Data obtained from Food Data Central [8]. PRAL in mEq/100g. Protein in g/100g. Calcium (Ca), Potassium (K), Magnesium (Mg) and Phosphorus (P) in mg/100g.
